# Supplementary material for: Evaluating teachers’ pedagogical content knowledge in implementing classroom-based assessment: A case study among esl secondary school teachers in Selangor, Malaysia
Source: PLoS One. 2023 Dec 29;18(12):e0293325. doi: 10.1371/journal.pone.0293325 (PMC10756533; doi:10.1371/journal.pone.0293325)
Supplement: Attachment — Submitted filename: Response to Reviewers.docx [file pone.0293325.s001.docx]

Reviewer #1: thank you for considering PLOS for you submission. This an interesting topic that aim to evaluating teachers’ pedagogical content knowledge in implementing classroom based Assessment I do believe the submission have a merit. However, there are a few suggestions that may increase the quality if the this paper.

1. The authors are suggested to formulate explicit hypotheses of the proposed relationships in figure 2. Further, these hypotheses should be theoretically supported.

We acknowledge the importance of theoretically supported hypotheses and ensure that they have included in our manuscript.

2. The discussion section can be enhanced by performing more comparisons between the findings of this study and the relevant literature.

We agree that enhancing the discussion section with more comparisons between our findings and the relevant literature would be beneficial. We have further explored and incorporated these comparisons to provide a more comprehensive analysis in our manuscript.

3. In the methodology section, it would be useful to have a paragraph to outline the process of data analysis.

We agree that providing a clear explanation of our data analysis procedures will enhance the transparency and reproducibility of our study. We have included a detailed description of the data analysis process in the revised manuscript.

Reviewer #2: This is a well-written research paper that has good contribution in the education field, particularly in Malaysia. In order to increase the quality of this paper, several aspects should be considered.

1. The introduction section of this paper lacks clear presentation of its main contributions. The authors should emphasize the research problems, significance, and gaps in the existing literature to effectively highlight their contributions.

We acknowledge the importance of emphasizing the research problems, significance, and gaps in the existing literature to effectively highlight our contributions. In the revised manuscript, we have provided a more explicit and focused presentation of our main contributions to address this concern.

2. In order to provide a comprehensive and organized research report, it is essential to present both theoretical and practical contributions separately following the discussion section. This separation allows for a clear distinction between the concepts and ideas put forth in the theoretical framework and the tangible outcomes observed in the practical implementation.

In the revised manuscript, we have done the separation of theoretical and practical contributions to enhance the clarity and organization of our research report.

3. While the analysis section includes the main elements of a good analysis, the multi-collinearity test is missed. Please perform and report.

We agree that this test is important to assess the presence of collinearity among the variables. In the revised manuscript, we have conducted a multi-collinearity test and reported the results to ensure a comprehensive analysis.

4. The introduction, discussion, and the proposed hypotheses can be enhanced by including recent and relevant literature:
- Pedagogical practices and challenges in cultivating moral values: A qualitative study of primary school teachers in Pakistan. Doi: https://doi.org/10.1080/03004279.2021.1992471
- Beliefs and Knowledge for Pre-Service Teachers’ Technology Integration during Teaching Practice: An Extended Theory of Planned Behavior Doi: https://doi.org/10.1080/07380569.2022.2124752
- Teachers’ Perceptions of Technology Integration in Teaching-Learning Practices: A Systematic Review Doi: https://doi.org/10.3389%2Ffpsyg.2022.920317
- Technology Integration in Higher Education During COVID-19: An Assessment of Online Teaching Competencies Through Technological Pedagogical Content Knowledge Model Doi: <https://doi.org/10.3389/fpsyg.2021.736522>

We appreciate your suggestion to include recent and relevant literature in the introduction, discussion, and proposed hypotheses. We have carefully reviewed the suggested articles and integrated relevant insights from them into our manuscript. By incorporating these references, we aim to enhance the scholarly foundation of our study and establish connections with the existing literature.
