# Supplementary material for: Evaluating teachers’ pedagogical content knowledge in implementing classroom-based assessment: A case study among esl secondary school teachers in Selangor, Malaysia
Source: PLoS One. 2023 Dec 29;18(12):e0293325. doi: 10.1371/journal.pone.0293325 (PMC10756533; doi:10.1371/journal.pone.0293325)
Supplement: Attachment — Submitted filename: Response to Reviewers (1).docx [file pone.0293325.s002.docx]

**Journal Requirements:**

**If you need to cite a retracted article, indicate the article’s retracted status in the References list and also include a citation and full reference for the retraction notice.**

We have clearly indicated the retracted status of the relevant articles in the References list of our manuscript. Additionally, we have provided citations and full references for the associated retraction notice as required.

**Additional Editor Comments:**

**However, I did notice that the paper still requires some proofreading. There are a few minor grammatical and typographical errors throughout the text that could be polished to enhance the overall readability of the paper. A careful proofreading pass or the assistance of a professional editor would be beneficial in this regard.**

We appreciate your suggestion to improve the readability of the manuscript through meticulous proofreading to correct minor grammatical and typographical errors present in the text. We have taken your advice to heart and have thoroughly proofread the entire manuscript. We have corrected all identified errors and have endeavoured to refine the language and presentation throughout to improve overall readability and coherence. We have also sought the assistance of a professional editor to ensure that no mistakes remain and to further polish the language. We believe these revisions have significantly improved the quality of the manuscript and have addressed the concerns you raised. We am hopeful that these modifications will meet your approval, and We are open to any further suggestions or corrections you may have.

**Reviewer #4:**

**The manuscript presents an interesting study. However it is advised that authors send the manuscript for professional proofreading, because there are some inconsistencies in the essay, and language style used. There are also references in text which were not cited in the Reference section. Authors are advised to not make "sweeping statements" about the generalisability of the findings, because the study only looked at one state in Malaysia.**

Thank you very much for your insightful comments and constructive feedback on our manuscript. We would like to confirm that we have engaged the services of a professional editor to proofread the manuscript thoroughly and address inconsistencies in the essay and language style, ensuring that the manuscript meets the high standard of quality expected of academic publications. We have carefully reviewed the manuscript to ensure that all references mentioned in the text are accurately and completely cited in the Reference section. We apologize for any oversight in the initial submission and appreciate your patience as we rectified this. We acknowledge your concern regarding the generalizability of our findings, given that our study focused solely on one state in Malaysia. In light of your advice, we have revised the relevant sections of the manuscript to clarify the scope and limitations of our study and have refrained from making sweeping statements about the generalizability of our findings. We now explicitly state the contextual boundaries of our results and emphasize the need for caution when extrapolating the results to other settings. We believe that these revisions have addressed your concerns effectively and have enhanced the overall quality of the manuscript.
